# Androstenedione and Follicle-Stimulating Hormone Concentration Predict the Progression of Frailty Syndrome at One Year Follow-Up in Patients with Localized Breast Cancer Treated with Aromatase Inhibitors

**DOI:** 10.3390/biomedicines10071634

**Published:** 2022-07-07

**Authors:** Javier García-Sánchez, Mayra Alejandra Mafla-España, María Dolores Torregrosa, Omar Cauli

**Affiliations:** 1 Medical Oncology Department, Doctor Peset University Hospital, 46017 Valencia, Spain; javier.garciasanchez@chwapi.be (J.G.-S.); torregrosa_dol@gva.es (M.D.T.); 2 Medical Oncology Department, Hospital Center of Wallonie Picardy, 7500 Tournai, Belgium; 3 Frailty Research Organized Group, University of Valencia, 46010 Valencia, Spain; maymaes@alumni.uv.es; 4 Department of Nursing, University of Valencia, 46010 Valencia, Spain

**Keywords:** aromatase inhibitors, letrozole, anastrozole, testosterone, gonadotropin, frailty, androgen, estrone, biomarker, androstenedione

## Abstract

Background: The standard treatment in postmenopausal women with estrogen- and progesterone-positive localized breast cancer consists of aromatase inhibitors (AROi). The ability of AROi to promote or worsen frailty syndrome over time and the relationship with changes in gonadal hormones concentration in blood have not been investigated. Methods: A prospective study to evaluate the relationship between frailty syndrome and gonadal hormones concentrations in blood at baseline (prior to AROi treatment) and after 6 and 12 months under AROi treatment in post-menopausal women with breast cancer. Frailty syndrome was evaluated by the Fried’ criteria. We evaluated whether hormone concentration at baseline could predict frailty syndrome at follow-up. Results: Multinomial regression analysis showed that of the different hormones, those significantly (*p* < 0.05) associated to the worsening of frailty syndrome were high androstenedione levels and low follicle-stimulating hormone (FSH) levels in blood. Receiver operating characteristic curve analysis showed both androstenedione and FSH significantly (*p* < 0.05) discriminate patients who developed or presented worsening of frailty syndrome over time, with acceptable sensitivity (approximately 80% in both cases) but low specificity (40%). Conclusion: Hormonal concentrations before AROi treatment constitute possible biomarkers to predict the progression of frailty syndrome.

## 1. Introduction

Breast cancer is a major health problem. It is estimated that approximately 1.2 million new cases are diagnosed worldwide each year, and that some 400,000 women die from the disease annually [1]. Breast cancer is already the most common cancer worldwide, accounting for 12% of all new annual cancer cases worldwide [2]. The figures in the specific case of Spain reflect an incidence corresponding to the year 2021 of 33,375 cases, with an estimated 6606 deaths for the year 2020 (data from the Global Cancer Observatory).

Currently, anti-estrogen therapies are the mainstay in the treatment of localized breast cancer characterized by high estrogen receptor (ER) positivity in tumor tissue. Given that the majority of breast cancers are ER positive, it can be said that pharmacologic therapies with estrogen synthesis inhibitors or estrogen receptor antagonist drugs have had a greater overall impact on the treatment of breast cancer [3]. Aromatase (which converts androstenedione to estrone and testosterone to estrogen) is the main source of estrogens in postmenopausal women, and its inhibitors lead to a reduction in estrogen levels (6). This action leaves women without residual estrogens, which are produced after menopause, such as residual estradiol and mainly estrone. In postmenopausal women, adjuvant hormonal therapy with anti-estrogens is based on the use, during at least 5 years, of third-generation aromatase inhibitors (AROi) such as anastrozole, letrozole and exemestane [4]. Recent clinical trials have shown AROi to be more effective than tamoxifen in reducing cancer recurrences [5].

As a consequence of the reduction of estrogen levels produced by such treatment, postmenopausal women are at a high risk of developing several side effects [6,7,8,9]. In this sense, frailty syndrome defined as a potentially reversible health condition characterized by a decrease in physiological reserve caused by impairment of multiple physiological systems [10], and leading to increased vulnerability to adverse health effects and decreased ability to react favorably to stressful events [10,11]. Menopause may have a predisposing role on the risk of frailty in older women, in fact a study of nearly 10,000 women aged 45–85 years showed that the age at menopause is inversely related to frailty syndrome [12].

Frailty was defined by Fried et al. as a state of high vulnerability to adverse health outcomes, including disability, dependence, falls, the need for long-term care and mortality [13,14,15]. Over the past two decades, studies have shown that the assessment of frailty syndrome is critical to health care and health outcomes, and is of particular importance for oncology patients undergoing various treatments such as surgery, chemotherapy and radiotherapy. Because both cancer per se as a disease and oncological treatments represent additional stress factors that reduce the patient’s physiological reserve, the incidence of frailty in cancer patients is especially high; in fact, it has been estimated that more than half of all elderly cancer patients suffer from frailty [16].

Hormonal changes that occur as a result of menopause may contribute to the onset or worsening of frailty syndrome. The onset of menopause leads to a decrease in estrogen accompanied by an increase in circulating gonadotropins concentration [6,17,18]. Several studies have suggested that marked fluctuations and changes in circulating estrogens and androgens contribute along with other biological mechanisms to the appearance of frailty through several basic aging mechanisms at cellular, tissue, organ and systemic levels [19]. A pioneering study on the relationship between estrogen concentration and inflammation has demonstrated a synergistic effect between high levels of estradiol and high levels of C-reactive protein, a ubiquitous marker of systemic inflammation, in the development of frailty syndrome [20]. In addition, a study in approximately 500 older women aged 70–79 years showed decreases in free testosterone to be a risk factor for frailty [14,21]. However, in women there are conflicting results regarding the relationship between testosterone levels and frailty [22,23], and the burden of androgens in blood has been shown to be a stronger predictor of frailty status although the relationship is nonlinear [24]. In a cross-sectional study, our group has shown that in postmenopausal women with ER-positive breast cancer, frailty syndrome is positively correlated to plasma estrone, gonadotropins concentrations and to the aromatase activity index in blood [23].

The identification of blood biomarkers associated with the progression of frailty syndrome over time may help clinicians understand the pathophysiological mechanisms of frailty, and facilitate the diagnosis and development of interventions to delay or reverse its progression over time. Longitudinal research studies allow estimation of a possible causal relationship that cannot be determined in cross-sectional studies. Distinguishing heterogeneity in different subgroups of patients is important for understanding individual variations between subjects as well as for developing predictors of adverse events over time related to frailty. In the present study, we have analysed the progression (over one year of follow-up) of frailty syndrome and its components defined in the physical phenotype of frailty syndrome in postmenopausal patients with breast cancer undergoing hormonal treatment with AROi. Since AROi treatment changes the concentration of estrogen, androgen and gonadotropin levels in blood, we have determined if these hormones can be used as peripheral biomarkers to predict the progression of frailty syndrome under AROi treatment at one year of follow-up.

## 2. Methods

### 2.1. Study Design and Participants

A longitudinal study was carried out in postmenopausal women with localized estrogen-positive breast cancers attended and followed-up on at the Oncology Department of a tertiary hospital (Dr. Peset University Hospital, Valencia, Spain).

The inclusion criteria were TNM stages T1-T2 breast cancer patients subjected to surgical treatment and radiation therapy with or without adjuvant chemotherapy, and who received hormone therapy with aromatase inhibitors (AROi) as adjuvant treatment to prevent cancer recurrence.

The exclusion criteria were the presence of cognitive impairment (Mini-Mental State Examination [MMSE] score < 24), blindness, poor understanding of the Spanish language, and the presence of metastatic disease.

The trial was conducted in accordance with the guidelines of the Declaration of Helsinki, and the study protocol was approved by the local Ethics Committee (CEIm Code Dr. Peset Hospital 57/18 date 7 August 2018). All participants gave written informed consent prior to enrollment in the study.

### 2.2. Study Variables

The study variables included sociodemographic characteristics (age, BMI, marital status, number of daily drugs consumed, Charlson comorbidity index), clinical variables of breast cancer (tumor type, histological type of tumor, time since diagnosis, type of AROi, TNM stage, radiotherapy, chemotherapy, and type of surgery). The age-adjusted Charlson comorbidity index was calculated with a free online number (https://www.mdcalc.com/charlson-comorbidity-index-cci) (accessed on 10 September 2021).

### 2.3. Measurement of Frailty Syndrome

The level of frailty was measured based on the Fried criteria [15], as follows: unintentional weight loss (5% or 4.5 kg or more in the last year); reported chronic fatigue: participants met the criterion if they answered “Always”, “Often”, or “Most of the time” to the question: “How often in the last week did you feel that everything you did was an effort?”, included in the Center for Epidemiological Studies Depression Scale; and low level of physical activity measured using the International Physical Activity Questionnaire (IPAQ), validated for the Spanish language. The total amount of energy expended in minutes in activities during one week was calculated and divided into percentiles. Individuals in the lowest percentile (less than 150 min/week) received a positive score for this frailty criterion.

According to the standards of physical performance and gait speed, participants who walked 4.6 m in a longer period of time were in the worst percentile considering the height of the participants, stratified as follows: women with height > 173 cm: ≥6 s; height < 173 cm: ≥7 s. To measure muscle weakness, the study established grip strength by quintiles, measured at baseline three times for each hand alternately, using a digital dynamometer (Jaymar, J.A. Preston, Corp., Jackson, MS, USA) according to the standards for Hispanic populations established by previous epidemiological studies in older people. Accordingly, all patients with a minimum score were considered to meet the criteria for frailty because of their decreased muscle strength. Participants were considered to be frail if they met at least three criteria, pre-frail if they met one or two criteria, and robust or non-frail if they did not meet any of the criteria. All measurements were performed by trained members of the Nursing Department of the University of Valencia (Valencia, Spain), using a questionnaire with detailed instructions.

### 2.4. Statistical Analysis

For the calculation of the minimum sample size (unicentric study) that was representative of the patients attended to in the Oncology Department of the Hospital Universitario Dr. Peset (Valencia, Spain), it was estimated that an average of 77 post-menopausal women with breast cancer started adjuvant treatment with aromatase inhibitors in the Hospital every year (with the program G*Power 3.1.9.2 (G*Power©, Dusseldorf, Germany) as described previously [23]. For the descriptive analysis, quantitative variables were presented with the mean and standard deviation (SD), and categorical variables with the absolute value and the corresponding percentage. For the bivariate analysis, the normal distribution of the quantitative variables was first calculated with the Shapiro-Wilk test in order to be able to use parametric or nonparametric tests. Correlation between quantitative variables was determined by Spearman’s correlation test (nonparametric test) or Pearson’s correlation test (parametric test). Differences between two groups were analyzed using the nonparametric Mann-Whitney U test or the parametric Student’s *t* test. Differences between three groups were analyzed using the nonparametric Kruskal-Wallis test, followed by post hoc tests. Multinomial logit regression models were used to assess the effects of variables found to be significant in the bivariate analysis for the risk of worsening frailty syndrome versus stable or improved frailty syndrome at 12-month follow-up. In order to assess the role of different hormones at baseline in the progression of frailty syndrome, a predictive model was designed for this outcome, including the variables of the univariate analysis at a significance level of 5%. In the longitudinal analysis, logistic regression was also used to examine associations between hormones and clinical endpoints relevant to worsening frailty syndrome such as patient age, previous chemotherapy, BMI, Charlson comorbidity index, or previous chemotherapy. Odds ratios (ORs), 95% confidence intervals (95% CIs) and statistical significance (*p*) were estimated for each predictor variable. The discrimination accuracy of the predictive model was calculated using the C statistic (area under the receiver operating characteristic curve; AUC). Statistical significance was set at *p* < 0.05. All statistical analyses were performed with the SPSS statistical package (version 24.0; SPSS, Inc., Chicago, IL, USA).

## 3. Results

### 3.1. Sociodemographic and Clinical Data

Forty-seven breast cancer patients were included at baseline and were followed-up for 12 months under AROi treatment. All patients had tumors with estrogen-and progesterone-positive staining in the histological tissue analysis. The patients presented TNM disease stages corresponding to stage I (2.1%), or stage II (72.1%) or stage III (25.5%). None of the patients presented distant metastases. All patients had received neoadjuvant treatment either with conservative surgery (91.6%) or mastectomy (8.4%). Some of them received adjuvant chemotherapy (*n* = 15; 31.9%) and radiotherapy (*n* = 44; 93.6%) as per protocol of their adjuvant treatment. One month after the last radiotherapy session, all women had started adjuvant hormonal treatment with aromatase inhibitors (anastrozole or letrozole) [23]. The sociodemographic and oncology data are shown in Table 1.

The hormonal treatment prescribed consisted of the AROi anastrozole (1 mg/day orally) or letrozole (2.5 mg/day orally).

### 3.2. Changes in Frailty Syndrome over Time under Treatment with Aromatase Inhibitors

**Figure 1:**
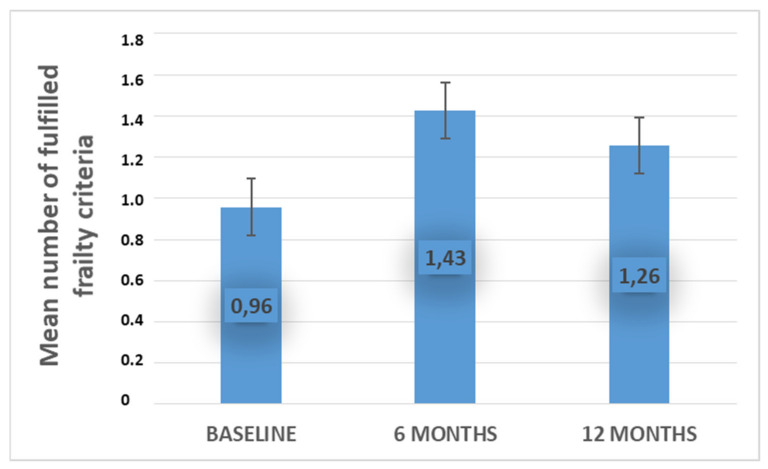
Changes in mean score of frailty syndrome criteria, before treatment (baseline), at 6 and 12 months of treatment with Aromatase Inhibitors.

The mean sum of frailty criteria at baseline was 0.96 ± 0.12 (SEM) (range 0–3), versus 1.43 ± 0.16 (SEM) (range 0–4) at 6 months of treatment, and 1.26 ± 0.15 (SEM) (range 0–4) at 12 months of treatment. A slight decrease in frailty syndrome was thus observed (Figure 1).

A total of 47.1% (*n* = 24) of the patients maintained a stable or improved level of frailty at 6 months of treatment with AROi (stable 29.5% and improvement in 17.6% of the cases), while 52.9% showed worsening of frailty syndrome. On comparing the results of the frailty criteria at baseline (i.e., before starting AROi administration) versus after one year of AROi treatment, an increase in the severity of frailty syndrome was observed in 23 women (48.9%). There was improvement of frailty during this period in 12 women (25.5%), i.e., their level of frailty decreased at one year with respect to baseline, while 12 women (25.5%) showed stable frailty levels between baseline and 12 months of AROi treatment.

The prevalences of the 5 frailty criteria at baseline (i.e., before starting drug treatment with AROi) and after 6 and 12 months under AROi treatment are shown in Table 1. There were significant differences over time in the prevalence of three of these criteria (Table 1). The percentage of women fulfilling the criterion related to self-perceived fatigue was 10.7% at baseline, and increased to 29.7% at 6 months and 27.6% at 12 months of follow-up (*p* = 0.04). In turn, the percentage of women fulfilling the criterion related to decreased physical activity was 31.9% at baseline, and increased to 40.3% at 6 months, followed by a decrease to 21.2% at 12 months under AROi treatment (*p* = 0.02). Lastly, the percentage of women fulfilling the criterion related to slow gait speed was 8.5% at baseline, and increased to 27.6% at 6 months and 27.6% at 12 months under AROi treatment (*p* = 0.001). No significant differences were observed for the other two frailty criteria, i.e., involuntary weight loss and diminished muscle strength (Table 2).

### 3.3. Relationship between Clinical Characteristics and the Progression of Frailty Syndrome

Logistic regression analysis can be used to evaluate the effects of several factors simultaneously that are likely to be related with the progression of frailty syndrome over time defined as an increase in the number of fulfilled frailty criteria at 12 months under AROi therapy versus the number of frailty criteria at baseline. Women without frailty syndrome progression were characterized by a stable number of frailty criteria under AROi treatment, or a decrease in the number of such criteria (i.e., decreased level of frailty syndrome), compared to the baseline level of frailty. The presence or not of progression of the frailty criteria at 12 months under AROi treatment was the dependent variable in the logistic regression analyses.

Logistic regression analysis was also used to determine the role of possible confounding factors such as age, previous chemotherapy treatment, polypharmacy, Charlson comorbidity index and BMI) in the development or progression of frailty syndrome over time (Table 3). On selecting the dichotomous variable presence (worsened frailty) or not (stable/improved frailty) as dependent variable, no significant effects were found with any of the aforementioned variables.

### 3.4. Changes in Gonadotropins, Androgens and Estrogens under AROi Treatment

The concentrations of gonadotropins and androgens, progesterone and estrogens, and the aromatase activity index in plasma at baseline, and after 6 and 12 months of AROi treatment are shown in Table 4.

No significant associations were observed between fatigue progression and the hormones FSH (*p* = 0.479, Mann-Whitney U-test), estradiol (*p* = 0.960, Mann-Whitney U-test), estrone (*p* = 0.290, Mann-Whitney U-test) or androstenedione (*p* = 0.611, Mann-Whitney U-test), and the aromatase activity index (*p* = 0.757, Mann-Whitney U-test).

The progression of gait speed was significantly associated to the hormones FSH (*p* = 0.021, Mann-Whitney U-test), estradiol (*p* = 0.023, Mann-Whitney U-test) and estrone (*p* = 0.042, Mann-Whitney U-test), but not to androstenedione (*p* = 0.715, Mann-Whitney U-test) or the aromatase activity index (*p* = 0.648, Mann-Whitney U-test)

### 3.5. Relationship between Androgen and Estrogen Concentrations in Blood and the Progression of Frailty Syndrome

**Figure 2:**
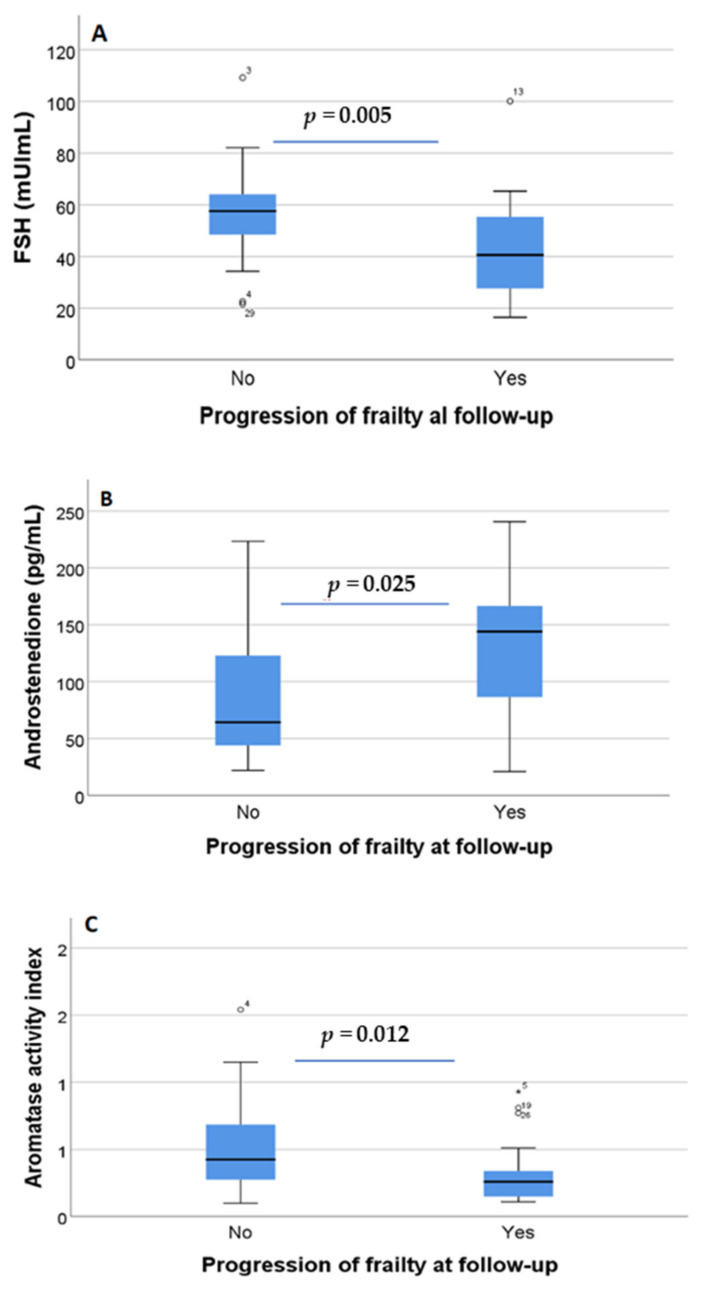
Differences in FSH (**A**), androstenedione (**B**) and aromatase activity index (**C**) between women with worsened frailty syndrome (progression) and those with stable or improved (no progression) frailty syndrome at 12 months under AROi treatment. On the boxplots shown in the Figure the outliers are identified (“out” values (small circle) and “far out” and “Extreme values” (marked with a star)).

On comparing the baseline concentrations of hormones in women showing progression of frailty syndrome at 12 months under AROi treatment versus those with stable or decreased frailty, no significant changes were observed for LH (*p* = 0.419), progesterone (*p* = 0.71), estrone (*p* = 0.337), testosterone (*p* = 0.567), dehydroepiandrosterone (*p* = 1.000) or dihydrotestosterone (*p* = 0.607). In contrast, there were significant changes in the concentration of FSH (*p* = 0.005) (Figure 2A) and androstenedione (*p* = 0.025) (Figure 2B), and in the aromatase activity index (*p* = 0.012) (Figure 2C) compared to baseline. Estradiol was detected at baseline in 12 women and it significantly decreased over time under AROi treatment (*p* = 0.007).

In the logistic regression analysis between the variable progression of frailty syndrome at 12 months under AROi treatment and a number of hormones at baseline, no significant associations were identified for many hormones (LH, estradiol, progesterone, testosterone, dehydroepiandrosterone sulfate, dihydrotestosterone and estrone). In contrast, on selecting as dependent variable the dichotomous variable worsened frailty and stable/improved frailty, significant effects were found for baseline androstenedione (*p* = 0.015; OR = 1.018, 95%CI = 1.004–1.032) and FSH (*p* = 0.034; OR = 0.954, 95%CI = 0.913–0.996) concentration, whereas the significant effect of the aromatase activity index at baseline was not significant in the logistic regression analysis model.

### 3.6. Diagnostic Sensitivity and Specificity of the Selected Biomarkers

**Figure 3:**
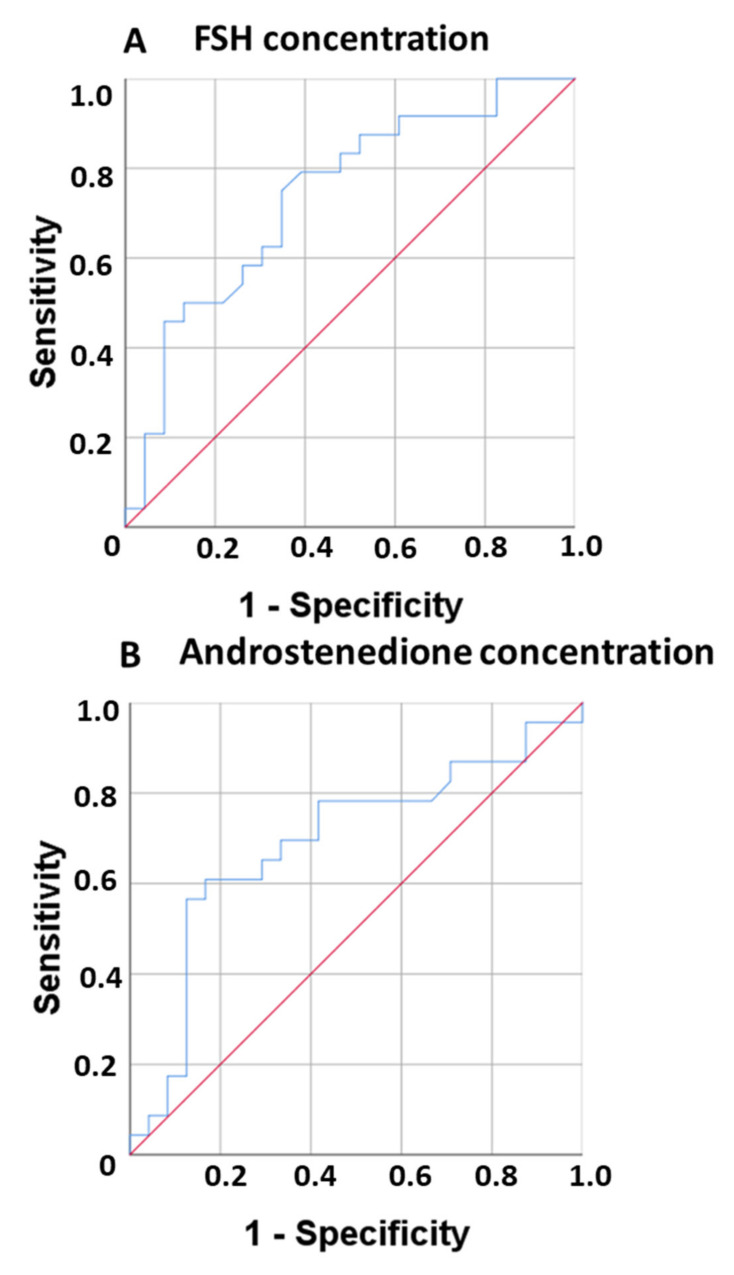
Receiver operating characteristic (ROC) curve for FSH (**A**) and androstenedione (**B**). The red line represent a hypothetical ROC curve of a perfect classifier. The blue curve represent the real ROC curve classifier.

We estimated the sensitivity and specificity of the two hormones at baseline that showed a significant effect in the logistic regression analysis in predicting the progression of frailty syndrome at 12 months under AROi treatment. A receiver operating characteristic (ROC) curve was plotted, since it is a useful tool for assessing the diagnostic potential of androstenedione and FSH at baseline in distinguishing between women with progression of frailty syndrome at 12 months under AROi treatment and those without progression (stable or decreased frailty syndrome). This analysis provided a comprehensive view of the sensitivity trend at all cut-off points, and thus provided information on the relationship between the sensitivity and specificity of FSH and androstenedione at baseline in differentiating between women with worsened frailty syndrome at 12 months under AROi treatment and those with stable or decreased frailty syndrome. The value of the area under the curve (AUC) for FSH was 0.737 (95% CI 0.593–0.881), with a cut-off point of 46.85, a sensitivity of 79.2% and a specificity of 39.1% (Figure 3A). The AUC for androstenedione was 0.691 (95% CI 0.532–0.850), with a cut-off point of 81.90, a sensitivity of 78.3% and a specificity of 41.7%. Lastly, the AUC for the aromatase activity index was 0.714 (95% CI 0.564–0.864), with a cut-off point of 29, a sensitivity of 70.8%, and a specificity of 34.8% (Figure 3B).

## 4. Discussion

To our knowledge, this longitudinal study is the first to analyze the evolution of frailty syndrome in postmenopausal women with estrogen receptor-positive breast cancer subjected to drug treatment with AROi. These drugs represent first line treatment in the adjuvant setting to prevent cancer relapse in these patients [25,26]. We have determined which components of frailty syndrome based on Fried’s physical phenotype worsened over time, which could lead in future studies to the opportunity to design multidisciplinary interventions to reduce the impact of them and thus to improve the quality of life of these patients. Notably, we have identified some gonadal hormones measured in blood that could help to identify those patients that develop or worsen frailty syndrome over time, in fact the evolution of frailty syndrome varied. Notably, almost 50% of the patients showed worsened frailty at 12 months of follow-up, while approximately 25% remained stable and 25% showed improvement of their frailty status. The latter effect (i.e., improvement) is not surprising, since frailty syndrome is a reversible condition mainly in the pre-frailty phase [27,28], and the women in our study were mostly (63.8%) prefrail (i.e., they met one or two frailty criteria) at baseline before receiving AROi treatment [23]. A recent longitudinal study (with one year of follow-up, as our study) in community-dwelling individuals found that one third of the frail older adults regressed to pre-frailty status, and 8.7% of the pre-frail older adults regressed to non-frailty status [29]. Regarding the clinical factors implicated in the worsening of frailty syndrome, we found no significant role in the regression analysis models on controlling for age, previous chemotherapy, number of daily drugs, Charlson comorbidity index and BMI on comparing patients with worsened versus non-worsened frailty status. These results were partially replicated in another study in community-dwelling individuals in Spain with no recent cancer history, in which the progression of frailty at 12 months of follow-up was only predicted by specific comorbidities present at baseline (congestive heart failure and hearing impairment) and polypharmacy [29]. Other factors apart from hormonal changes have been linked to frailty syndrome, evidencing a clear multifactorial pathogenesis [30,31]. For instance, genetic factors were recently investigated in a study among frail community-dwelling individuals in Spain. Genetic susceptibility for frailty syndrome was found at the level of 15 single nucleotide polymorphisms (SNPs), 18 genes, and four pathways related to cytokine-cytokine receptor interaction, natural killer cell-mediated cytotoxicity, the regulation of autophagia, and the renin-angiotensin system as the factors most strongly associated to frailty syndrome [32]. Future studies should explore the effect of AROi upon these genetic changes in breast cancer patients, in order to stratify individuals with an increased risk of developing frailty or of suffering a worsening of frailty during AROi treatment. This is crucial, since the current oncology guidelines indicate at least 5 years of treatment with AROi in the adjuvant setting, and frailty syndrome in older women with breast cancer is associated to a higher risk of all-cause mortality, but not of breast cancer-specific mortality [33]. Regarding the individual component of frailty syndrome based on the physical phenotype of frailty [15], we observed significant worsening under AROi treatment in relation to two criteria, namely self-reported fatigue and gait speed. Few data are available on the prevalence of fatigue among patients receiving anti-estrogenic treatment for breast cancer, though a relationship has been demonstrated between the use of the estrogen receptor antagonist tamoxifen and AROi and the occurrence of self-reported fatigue [34,35]. Gait speed is dependent on both muscular and neural factors, so it is conceivable that decreased estrogen production under iARO treatment may have an initial impact on neuromuscular unit performance. Interestingly, a previous study on the effects of the administration of the AROi anastrozole in men with low testosterone levels also recorded a decrease in gait speed [36]. However, there is discrepancy between studies in frail older people regarding the association between estrogen levels and physical performance, including walking speed [37]. Our findings suggest that reduced levels of estrogen are unfavorable for physical performance as measured by gait speed, probably due to an effect upon skeletal muscle [38].

Changes in peripheral androgen and estrogen play an important role in the onset, recurrence and progression of the majority of breast cancers [39], and also in the pathophysiology of frailty syndrome [40,41,42,43], along with other factors such as inflammation and metabolic disorders [44,45,46]. The identification of biomarkers that could help clinicians to identify patients that develop frailty syndrome or suffer worsening of frail syndrome would facilitate clinical decision making and reduce adverse outcomes related to frailty [47,48,49]. As expected, during AROi treatment the concentration of estrogen in blood decreased due to aromatase inhibition [50,51], as evidenced in our study by the decrease in the aromatase activity index, the reduction of estrone (the main estrogen in postmenopausal women) [52,53] and the increase in androstenedione (the precursor of estrone) [53,54]. We also observed a significant increase over time in the concentration of the gonadotropin FSH during AROi treatment, as previously reported [55,56].

Our results show that some hormonal changes at baseline, such as an increase in androstenedione and FSH in blood, identified those women with progression of frailty syndrome at 12 months under AROi. It is plausible that increased androstenedione concentrations in blood at baseline could be due to reduced aromatase activity, as demonstrated in our study, though the aromatase activity index was not significantly associated with the progression of frailty syndrome in the regression models. The rise in androstenedione concentration could also be due to an increase in the production of the hormone in a subgroup of women with breast cancer who suffered worsened frailty syndrome at follow-up. In fact, our longitudinal study recorded an increase in androstenedione concentration and a decrease in estrone levels at 12 months of follow-up under AROi treatment, suggesting that androstenedione could be a direct or indirect hormonal factor involved in the worsening of frailty syndrome. The source(s) of the increase in androstenedione production in such women is not known, though several hypotheses could be suggested. In the postmenopausal state, the ovaries lose aromatase enzyme, though they continue to secrete androstenedione [57]; one source of androstenedione therefore could be the ovaries [58]. Androstenedione concentration has been previously reported to be inversely associated to FSH concentration [59]. In our study, the FSH levels in blood significantly increased under AROi treatment, and lower FSH concentrations at baseline predicted a worsening of frailty syndrome over follow-up under AROi treatment. The role of FSH in frailty syndrome has not been investigated in humans, but it seems to be related to bone and anthropometric alterations also found in frailty syndrome. For instance, in animal models, blockade of FSH action increases bone mass [60,61,62] and decreases fat mass [60,63,64], and it has been postulated that increased circulating FSH levels may be another strong factor mediating the decrease in skeletal muscle mass during the menopausal transition [64]. In a population-based study of older postmenopausal women, it was shown that those with higher gonadotropin FSH values (the highest quartile) had lower bone mineral density, lower body weight, less visceral adipose tissue, but also less lean mass [65]. Other sources of increased androstenedione production could also be involved, such as the adrenal glands and peripheral tissues [66,67]. In the postmenopausal state, the adrenal glands are the main producers of aromatase substrates through direct secretion of the androgen testosterone and androstenedione [54,68]. In addition, dehydroepiandrosterone and its sulfate congener are secreted by the adrenal glands and converted into the aromatase substrates androstenedione and testosterone, in peripheral tissues and particularly in fat and skeletal muscle [69,70]. However, the fact that testosterone was not associated to frailty or to the progression of frailty under AROi treatment suggests that this hypothesis of general androgen production is unlikely. The increase in androstenedione was not associated to a parallel increase in testosterone concentration at baseline in women with worsened frailty at follow-up. These results agree with the main conclusion of a recent meta-analysis which found that testosterone levels in blood are not associated to frailty syndrome in women [41]. The results obtained in relation to androstenedione and FSH, after being confirmed in a larger group of postmenopausal women with breast cancer, could represent the basis for stratifying the risk of onset or worsening of frailty under AROi treatment, and may afford a useful biomarker for monitoring intervention strategies in these patients. A deep learning model based on a multiomic-based hormones panel [71] will be able to classify women who progress in frailty syndrome over time and could be useful to tailor personalized treatments and interventions as it has been suggested for survival rates in longer follow-up studies in women with breast cancer [72]. A better understanding of the pathophysiological basis of hormonal changes associated with improving or maintaining frailty in women with breast cancer would allow early risk factors to be determined and identify those individuals who are most likely to benefit from specific interventions to reduce or prevent the progression of the frailty syndrome and therefore the adverse effects on health that it determines [47,73]. The present study has some important limitations that should be highlighted. Firstly, the study was unicentric and the sample size, although representative for the hospital’s medical oncology service, is too small to evaluate the impact of specific clinical variables such as the role of comorbidities or polypharmacy in the development of frailty in the follow-up. Secondly, although the frailty syndrome has been defined according to Fried’s universally accepted criteria for the description of the physical phenotype of frailty, it must be remembered that the frailty syndrome has been conceptualized as a multidimensional geriatric syndrome that is mediated by important psychological and social variables that should be evaluated in future studies. However, in our view, the Fried’s criteria have several advantages that make them suitable for the clinical setting, as they are objective, brief and easy to use. We were unable to satisfactorily assess the impact of previous chemotherapy regimens as we had only 8 women who had received adjuvant chemotherapy and future studies need to compare the impact of the previous chemotherapy on the progression of frailty syndrome in case-control studies with/without chemotherapy and with/without hormonotherapy in patients with breast cancer.

## 5. Conclusions

Hormonal therapy after the first year of adjuvant treatment for breast cancer with aromatase inhibitors induces or increases the severity of frailty syndrome in postmenopausal women. Almost half of them have accumulated more frailty criteria at follow-up of 12 months. Among this group of women, lower blood levels of FSH and higher blood levels of androstenedione have been detected compared to women with stable or improved frailty criteria over this time. To date, these biomolecules represent the first two biomarkers able to discriminate women who developed or experienced worsened frailty syndrome under aromatase inhibitor treatment. More studies with a larger sample are required to confirm the sensitivity and specificity of these biomarkers and longer follow-ups are necessary to estimate this potential reversible or treatable side effect induced by these drugs. This will allow devising preventive actions in order to prevent the appearance of disability that could lead to a lack of adherence to adjuvant treatment and greater comorbidity.

## Figures and Tables

**Table 1:** Sociodemographic and clinical characteristics.

Variables	Frequency% (Categorical Variables) or Mean and Standard Error of the Mean (Range Min-Max) (Discrete Variables)
Age (years)	66.8 ± 1.3 (52–83)
Marital status:	
Married	23 (48.9%)
Divorced	7 (14.9%)
Separated	2 (4.3%)
Single	4 (8.5%)
Widow	11 (24.4%)
Histology of tumor:	
Ductal carcinoma	46 (97.9%)
Lobular carcinoma	1 (2.1%)
Estrogen receptor staining (%)	93.1 ± 1.6 (40–100)
Progesterone receptor staining (%)	61.3 ± 0.09 (1–3)
HER2-positive staining (patients with 3 + staining in HER2: 4 patients)	2.5 ± 5.2 (0–10)
Ki67 mean values (%)	15.4 ± 2.03 (1–60)
Previous chemotherapy	
Yes	8 (17.0%)
No	39 (83.0%)
Previous radiotherapy	
Yes	44 (93.6%)
No	3 (6.4%)
Charlson comorbidity index	2.5 ± 0.1 (2–5)
Body mass index	28.9 ± 0.8 (18.7–45)

**Table 2:** Prevalence of frailty syndrome criteria at baseline and under AROi treatment.

	Baseline	6 Months	12 Months	*p*-Value
Weight loss	Yes 11 (23.4%	Yes 9 (19.2%)	Yes 9 (19.1%)	*p* = 0.9
	No 36 (76.6%)	No 38 (80.8%)	No 38 (80.9%)	
Fatigue	Yes 5 (10.7%)	Yes 14 (29.7%)	Yes 13 (27.6%)	*p* = 0.04
	No 42 (89.3%)	No 33 (70.3%)	No 34 (72.3%)	
Physical activity	Yes 18 (35.3%)	Yes 23 (45.1%)	Yes 21 (41.2%)	*p* = 0.438
	No 29 (56.9%)	No 24 (47.1%)	No 26 (51.0%)	
Gait speed	Yes 4 (8.5%)	Yes 13 (27.6%)	Yes 17 (36.2%)	*p* = 0.001
	No 43 (78.7%)	No 34 (72.3%)	No 30 (63.8%)	
Muscle strength	Yes 10 (21.3%)	Yes 14 (29.8%)	Yes 12 (25.5%)	*p* = 0.7
	No 37 (78.7%)	No 33 (70.2%)	No 35 (74.5%)	

**Table 3:** Logistic regression model: clinical variables associated to the outcome variable (progression or not of frailty syndrome at follow-up).

Variables	*p*-Value	OR	95% CI
Age	0.257	1.048	0.967–1.135
Chemotherapy	0.728	1.354	0.246–7.458
Number of daily drugs	0.934	0.986	0.703–1.383
Charlson comorbidity index	0.181	2.140	0.702–6.520
Body mass index	0.337	1.067	0.934–1.219

**Table 4:** Concentration of gonadotropins and androgens, progesterone and estrogens, and aromatase activity index in plasma.

Hormone Concentration in Plasma	Baseline	6 Months of AROi Treatment	12 Months of AROi Treatment	*p*-Value
FSH (mIU/mL)	49.4 ± 2.93	52.31 ± 3.46	52.33 ± 3.54	0.01
LH (mIU/mL)	20.27 ± 1.25	20.5 ± 1.31	19.9 ± 1.59	0.92
Progesterone (ng/mL)	0.07 ± 0.03	0.08 ± 0.05	0.10 ± 0.08	0.62
Estrone (pg/mL)	31.3 ± 1.97	24.3 ± 1.4	24.1 ± 1.3	0.001
Estradiol (pg/mL)	7.57 ± 0.80	5.30 ± 0.21	5.9 ± 0.39	0.007
Testosterone (ng/mL)	0.27 ± 0.02	0.26 ± 0.02	0.27 ± 0.03	0.19
Dehydroepiandrosterone (µg/dL)	92.3 ± 8.16	83.3 ± 7.54	83.9 ± 7.71	0.57
Androstenedione (pg/mL)	108.4 ± 9.0	111.8 ± 7.8	117.9 ± 8.3	0.01
Dihydrotestosterone (ng/mL)	0.14 ± 0.009	0.15 ± 0.12	0.13 ± 0.10	0.36
Aromatase activity index	0.41 ± 0.05	0.26 ± 0.02	0.25 ± 0.02	0.001

## Data Availability

The data presented in this study are available on request from the corresponding author for scientific purposes.
